# Particle Size-Dependent Monthly Variation of Pollution Load, Ecological Risk, and Sources of Heavy Metals in Road Dust in Beijing, China

**DOI:** 10.3390/toxics13010040

**Published:** 2025-01-07

**Authors:** Cong Men, Donghui Li, Yunqi Jing, Ke Xiong, Jiayao Liu, Shikun Cheng, Zifu Li

**Affiliations:** Beijing Key Laboratory of Resource-Oriented Treatment of Industrial Pollutants, School of Energy and Environmental Engineering, University of Science and Technology Beijing, Beijing 100083, China; mencong@ustb.edu.cn (C.M.);

**Keywords:** heavy metal, road dust, source apportionment, temporal variation, risk assessment

## Abstract

Road dust carries various contaminants and causes urban non-point source pollution in waterbodies through runoff. Road dust samples were collected in each month in two years and then sieved into five particle size fractions. The concentrations of ten heavy metals (As, Cd, Cr, Cu, Hg, Mn, Ni, Pb, Zn, Fe) in each fraction were measured. The particle size fraction load index, coefficient of divergence, and Nemerow integrated risk index were used to analyze the temporal variation of pollution load and ecological risk in different particle size fractions. The advanced three-way model and wavelet analysis were used in quantitative identification and time-series analysis of sources. Results showed that both the pollution load and ecological risk of most heavy metals showed a decreasing trend from the finest fraction (P1) to the coarsest fraction (P5). The frequency of heavy metals in P1 posing extreme risk was about two times that of P5. Main types of heavy metal sources were similar among different fractions, whereas the impact intensity of these sources varied among different fractions. Traffic exhaust tended to accumulate in finer particles, and its contribution to Cu in P5 was only 35–55% of that in other fractions. Construction contributed more to coarser particles, and its contribution to Pb was increased from 45.34% in P1 to 65.35% in P5. Wavelet analysis indicated that traffic exhaust showed periodicities of 5–8 and 10–13 months. Fuel combustion displayed the strongest periodicity of 12–15 months, peaking in winter.

## 1. Introduction

Road dust is the source and sink of various pollutants, affecting the quality of waterbodies [1]. Pollutants in road dust may be dispersed into road runoff and then enter waterbodies, aggravating contamination in waterbodies [2]. Although the particle size range of road dust was not uniform among different studies, existing studies all showed that the migration of road dust and the enrichment of pollutants in it are both influenced by the particle size distribution [3,4]. Road dust particles less than 100 μm can be transported into the atmosphere under the influence of wind and other air currents. The diffusion ability of road dust particles between 100 μm and 150 μm is weaker than those of suspension. Road dust particles with a particle size greater than 150 μm have the weakest diffusion ability and mainly creep along the ground. Under the same rainfall washout condition, the migration of finer particles (<250 μm) is also significantly stronger than coarser particles (>250 μm) [5]. Of the road dust entering surface runoff, road dust smaller than 250 μm accounted for about 80% of the overall pollutant load, and road dust smaller than 44 μm accounted for about 70%. Therefore, in order to comprehensively understand the impact of road dust, attention should be paid to its particle size distribution.

Heavy metals in road dust can disrupt the ecological system and threaten human health. They can reach a level of critical concentration through bioaccumulation along the food chain [6]. Heavy metals can impair the nervous system, respiratory system, and endocrine system and can also destroy DNA and reduce the survival rate of organisms [7,8]. Nemerow integrated pollution index (*NIPI*) and potential ecological risk index (*RI*) have been widely used in pollution and risk assessment of heavy metals [9]. However, the *NIPI* neglects the differences of toxic response factors, which differ dozens of times between some heavy metals [10,11]. *RI* takes toxic response factors into account, but the result may be enlarged with increased numbers of assessed species for a certain sample [12]. In our previous study, a new method named Nemerow integrated risk index (*NIRI*) was developed to assess the integrated ecological risk of heavy metals based on the *NIPI* and *RI* [13]. The *NIRI* overcomes the defects of the *NIPI* and *RI*, which include the influence of the number of heavy metals on the value of the *RI* and the lack of consideration of the difference in toxic response factors of heavy metals in the evaluation of the *NIPI*. The *NIRI* can provide a more accurate evaluation of the integrated effect of multiple types of heavy metals. It also introduces the toxic response factor to differentiate the effects of heavy metals. To have a better understanding of ecological risk associated with road dust and increase the comparability of risk assessment among different studies, *NIRI* should be adopted to analyze the ecological risk associated with heavy metals in road dust. The long time series analysis on ecological risk of heavy metals can also help obtain a more reliable and comprehensive understanding of the real situation of ecological risk.

Quantitative risk apportionment of heavy metals in road dust is beneficial to effective control of ecological risks [14]. The positive matrix factorization (PMF) model has been widely used in source apportionment of heavy metals [15,16,17]. Compared with other models, such as principal component analysis, the PMF model can better reflect reality by implementing non-negativity constraints [18]. However, as a two-way source apportionment model, the PMF model can deal with source apportionment problems with two-way datasets (heavy metals and sampling time), and concentrations of heavy metals in different particle size fractions sampled at different times could not be inputted in the model for each run [16,19]. To obtain more information through source apportionment, the advanced three-way model (ABB three-way model) was developed, permitting the extraction of maximal information from three-way datasets [20]. Compared with the PMF model, results based on the ABB three-way model were more consistent with true conditions [21]. Existing studies focused on the source apportionment of road dust were based on two-way source apportionment models other than the ABB three-way model. The application of the ABB three-way model in source apportionment can help address real conditions and take full advantage of three-way dataset information (heavy metals, sampling time, and particle size fractions) [22].

Beijing is a metropolis under the influence of intense human activities. In this study, based on sifted road dust samples in Beijing over 24 months, the monthly variation of particle size fraction load, ecological risk, and sources of heavy metals in road dust were explored. The main objectives in this study were to (1) characterize the correlation and differences of load for heavy metals among different particle size fractions; (2) evaluate the monthly variation of single and integrated ecological risk associated with heavy metals within different particle size fractions; and (3) quantitatively identify the main sources of heavy metals in each fraction and analyze the time-series variation of impact intensity of these sources. These results will provide guidance for the adoption of management strategies that should vary in time and focus on the most influential source of heavy metals. The research framework is also referential for studies focused on ecological risk and sources on a city scale.

## 2. Materials and Methods

### 2.1. Study Area and Road Dust Sampling

**Figure 1:**
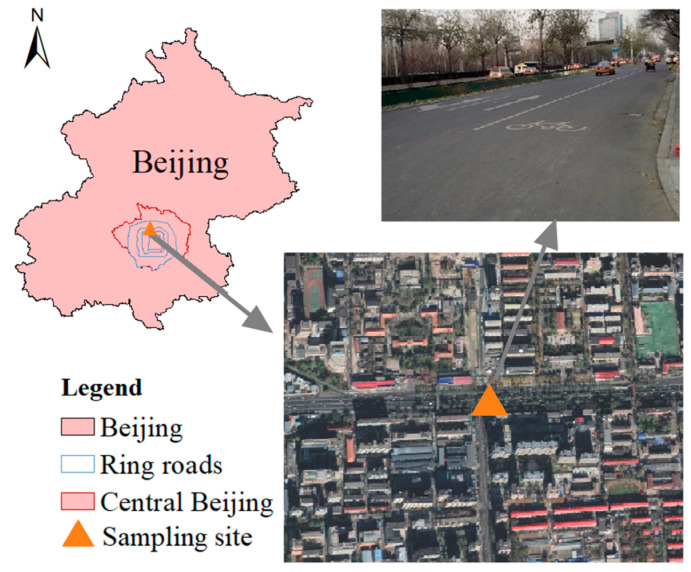
The study area and sampling site.

Beijing (115.7–117.4° E, 39.4–41.6° N), the capital of China, holds a significant position due to its roles in politics, culture, international relations, and technological innovation (Figure 1). Beijing is situated on the northwestern edge of the North China Plain. The city is in a warm temperate, semi-humid, continental monsoon climate, with four distinct seasons [23]. The summer is dry and cold; the winter is hot and rainy. By 2012, Beijing’s urbanization rate had reached 86.20%, further increasing to 86.5% by 2018 [24]. As of the end of 2023, Beijing’s permanent population reached 21.858 million. The demands of such a large population result in substantial resources and energy consumption, leading to the contamination of various pollutants and posing potential risks [25].

Road dust samples were collected monthly on the North Third Ring Road, Beijing, from May 2016 to April 2018, with a total of 24 sampling events (Figure 1). The sampling site was located in the urbanized area, which has been built for dozens of years. This area was densely populated and under the strong impact of human activities. Our previous study showed that this site was under heavier heavy metal contamination than most areas in Beijing [13]. To minimize the sampling bias over the 24-month sampling period, strategies were adopted: (1) samples were collected at the beginning of each month, after the ground had been dry for more than seven days, with no rainfall during this period; no rainfall occurred during any of the sampling events; all samplings were carried out on windless weekdays; and the same hardened surface was selected and carefully cleaned with a clean brush during each sampling. The collected dust was transferred into clean polyethylene self-sealing bags using a clean plastic scoop. More than 100 g of road dust was collected from each site. The samples were then transported to the laboratory for preprocessing. In the laboratory, clean nylon sieves (with mesh openings of 1000 μm) were used to remove debris such as cigarette butts and plant fragments. A portion of the dust with particle sizes smaller than 1000 μm from each sampling point was retained for further experimental use.

### 2.2. Analytical Methods and Quality Control

The pre-treated samples were further sieved, and the mass of road dust corresponding to each particle size class was recorded using nylon vibrating sieves instead of alloy vibrating sieves, and the mesh sizes were 35 mesh, 50 mesh, 80 mesh, 140 mesh, and 270 mesh, respectively. Five different particle size fractions of road dust were obtained; they were P1 (0–74 μm), P2 (74–125 μm), P3 (125–250 μm), P4 (250–500 μm), and P5 (500–1000 μm).

The heavy metal concentration in road dust samples from different particle size fractions was determined in accordance with experimental standards. The analyzed heavy metals included arsenic (As), cadmium (Cd), chromium (Cr), copper (Cu), mercury (Hg), manganese (Mn), nickel (Ni), lead (Pb), zinc (Zn), and iron (Fe). The concentrations of Cd, Cu, Ni, Pb, and Zn were quantified by inductively coupled plasma mass spectrometry (ICP-MS). The concentrations of Cr, Mn, and Fe were analyzed using inductively coupled plasma optical emission spectrometry (ICP-OES). The concentrations of As and Hg were determined by hydride generation atomic fluorescence spectrometry (HG-AFS) and cold vapor atomic fluorescence spectrometry (CV-AFS), respectively. The detection limits for As, Cd, Cr, Cu, Hg, Mn, Ni, Pb, Zn, and Fe were 1 μg/g, 0.02 μg/g, 2 μg/g, 1 μg/g, 2 μg/g, 10 μg/g, 2 μg/g, 2 μg/g, 2 μg/g, and 1000 μg/g, respectively. To ensure the reliability of the experimental results, certified reference materials, blank samples, and duplicate samples were used during measurements. No heavy metals were detected in blank samples, and the recovery rate was within ±10%. The reproducibility of duplicate samples was 100%. These findings confirm the high reliability of the heavy metal concentration measurements in this study, which can be used for subsequent analysis.

### 2.3. Particle Size Fraction Load Index

To determine the load of heavy metals in each particle size fraction, defined as the contribution of heavy metals in each fraction relative to the total heavy metal concentration in the road dust, the particle size fraction load (%*PSF_Load_*) index was used in this study [26]. In calculating the heavy metal load using this index, both the heavy metal concentration in each particle size fraction and the corresponding mass of road dust were considered. The formula is as follows:

(1) $$\%PSF_{Load}=\frac{C_{i}\times PS_{i}}{\sum_{i=1}^{n}C_{i}\times PS_{i}}\times 100\%$$

where *C_i_* represents the heavy metal concentration in particle size fraction *i*; *PS_i_* refers to the mass fraction of particle size *i* relative to the total mass of road dust samples; and *n* denotes the number of particle size fractions.

SPSS v.20 and one-way analysis of variance (ANOVA) were used to compare the values of %*PSF_Load_* among different particle size fractions in this study.

### 2.4. Coefficient of Divergence

The coefficient of divergence (CD) is widely used in measuring the similarity of pollutants under two conditions. In this study, CD was used to analyze the correlation of heavy metal loadings between different particle size fractions. The formula is as follows [27]:

(2) $$CD_{kl}=\sqrt{\frac{1}{p}{\left(\sum_{j=1}^{p}\frac{\%PSF_{Load}^{jk}-\%PSF_{Load}^{jl}}{\%PSF_{Load}^{jk}+\%PSF_{Load}^{jl}}\right)}^{2}}$$

where *CD_kl_* represents the coefficient of variation of heavy metal loads between particle size fractions *k* and *l*, and $\%PSF_{Load}^{jk}$ and $\%PSF_{Load}^{jl}$ represent the heavy metal loads of metal *j* in particle size fractions *k* and *l*, respectively. The parameter *p* denotes the number of heavy metal types, which is 10 in this study. Pairwise comparisons were made between particle size fractions, resulting in a total of ten CD values. If the CD value is less than 0.269, the two datasets are considered to be similar [27,28]. If the CD value is higher than 0.269, it means that differences exist between the two datasets.

### 2.5. Ecological Risk and Pollution Assessment Methods

The Nemerow integrated risk index (*NIRI*) was used in ecological risk assessment in this study. *NIRI* was developed in our previous study by combining two widely used assessment methods: potential ecological risk index (*RI*) and Nemerow integrated pollution index (*NIPI*) [13]. *RI* considers the integrated effect of multiple heavy metals, whereas the number of heavy metals considered has a significant effect on the evaluation results. The evaluation results of *NIPI* are not affected by the number of heavy metals considered, whereas the method does not take into account differences in toxicity among different heavy metals. *NIRI* takes into account the toxicity response coefficients of different heavy metals, and the evaluation results of *NIRI* are not significantly affected by the number of heavy metals. Therefore, the *NIRI* can obtain more reasonable results than *NIPI*, and assessment results based on *NIRI* are more comparable among different studies. The formula of *NIRI* is as follows:

(3) $$NIRI=\sqrt{\left(E_{r\max}^{i2}+E_{r\mathrm{ave}}^{i2}\right)/2}$$

(4) $$E_{r}^{i}=T_{r}^{i}\times C_{i}/S_{i}$$

where $E_{r}^{i}$ represents the potential ecological risk index of heavy metal *i*; $E_{r\max}^{i}$ and $E_{r\mathrm{ave}}^{i}$ are the maximum and average values of $E_{r}^{i}$ among all heavy metals, respectively; *C_i_* and *S_i_* are the concentration of heavy metal *i* measured and the corresponding reference background value for heavy metal *i*, expressed in μg/g in this study; and $T_{r}^{i}$ is the toxicity response coefficient of heavy metal *i*. The classification standards for *NIRI* are shown in Table 1. In this study, the ecological risks associated with As, Cd, Cr, Cu, Hg, Mn, Ni, Pb, Zn, and Fe were assessed, with the *T_r_* values for each metal being 10, 30, 2, 5, 40, 1, 5, 5, 1, and 1, respectively [29]. The background values of heavy metal concentrations in road dust were derived from the soil background values of Beijing, which is the earliest soil survey result [30]. The background values for As, Cd, Cr, Cu, Hg, Mn, Ni, Pb, Zn, and Fe are as follows: 9.6 μg/g, 0.079 μg/g, 57.3 μg/g, 20.7 μg/g, 0.038 μg/g, 540 μg/g, 24.9 μg/g, 23.5 μg/g, 68.0 μg/g, and 2.97%, respectively.

The formula of *NIPI* is as follows:

(5) $$NIPI=\sqrt{\left(PI_{i\mathrm{ave}}^{2}+PI_{i\max}^{2}\right)/2}$$

(6) $$PI_{i}=C_{i}/S_{i}$$

where *PI_i_* represents the pollution index of heavy metal *i*; *PI_ima_*_x_ and *PI_iave_* are the maximum and average values of *PI_i_* among all heavy metals, respectively. *C_i_* and *S_i_* are the concentration of heavy metal *i* measured and the corresponding reference background value for heavy metal *i*.

### 2.6. Advanced Three-Way Model (ABB Three-Way Model)

For the source analysis of the three-way datasets (elements, sampling time, and particle size fractions), the temporal change of source contributions is consistent among different particle size fractions, whereas source profiles might differ among different particle size fractions. Based on the characteristics of source contributions and source profiles [20], a three-way source analysis model was developed; the formula is as follows:

(7) $$x_{ijk}=\sum_{h=1}^{H}a_{ih}b_{jhk}+e_{ijk}$$

where *x_ijk_* represents the concentration of the *j*th element in the *k*th particle size fraction of the *i*th sample (the sample collected in the *i*th month); *a_ih_* denotes the contribution of the *h*th source to the *i*th sample; *b_jhk_* is the concentration of the *j*th element in the *p*th source of the *k*th particle size fraction; *e_ijk_* represents the residual; and *H* refers to the number of sources.

In the ABB three-way model, the source contribution matrix across different particle size fractions is unique and is denoted by *a_i__h_*, and the source composition matrix is non-unique and represented by *b_j__hk_*, which is the reason why the model is referred to as the ABB three-way model.

The ABB three-way model applies non-negative constraints to both the source contribution and source composition matrices, and it also assigns weights to account for the uncertainty of each data as follows:

(8) $$S_{ijk}=\left\{\begin{array}{c} \frac{5}{6}\times MDL\\ \sqrt{{\left(RSD\times x_{ijk}\right)}^{2}+{\left(0.5\times MDL\right)}^{2}} \end{array}\begin{array}{c} x_{ijk}<MDL\\ x_{ijk}>MDL \end{array}\right.$$

where *S_ijk_* denotes the uncertainty associated with the concentration of the *j*th element in the *k*th particle size fraction of the *i*th sample; *x_ijk_* represents the concentration of the *j*th element in the *k*th particle size fraction of the *i*th sample; *MDL* refers to the method detection limit; and *RSD* denotes the relative standard deviation of the measured element concentration.

The *Q* value is one of the judgment criteria of the source apportionment model, which is expressed by the following formula:

(9) $$Q={\sum_{i=1}^{n}\sum_{j=1}^{m}\sum_{k=1}^{r}\left[\frac{x_{ijk}-\sum_{h=1}^{H}a_{ih}b_{jhk}}{S_{ijk}}\right]}^{2}$$

where *r* denotes the total number of particle size fractions, *m* indicates the number of elements involved in the source apportionment, and *n* represents the total number of samples. The meanings of the other parameters are consistent with those in Formulas (7) and (8).

The ABB three-way model was run based on Multilinear Engine 2, and the number of runs was set to 200 times in this study. In the source apportionment based on the ABB three-way model, sources were determined based on both representative heavy metals in each particle size fraction, literature introducing heavy metal compositions in different sources, and the information collected in the study area.

### 2.7. Wavelet Analysis

Wavelet analysis shows multi-resolution capabilities in both time and frequency domains, which allows for the examination of variations across different time scales [31]. The principle behind wavelet analysis is to express a function in terms of basic wavelet functions. The wavelet function is the base for wavelet analysis, and the wavelet function $\Psi (t)\in L^{2}(R)$ must satisfy the following formula:

(10) $$\int_{-\infty }^{+\infty }\Psi (t)\mathrm{dt}=0$$

where Ψ(t) represents the base wavelet function, which can be used to generate a wavelet system through scaling and translation [32].

(11) $$\Psi_{a,b}(t)={\left|a\right|}^{-1/2}\Psi (\frac{t-b}{a}),\;a,b\in R,\;a\;\neq \;0$$

where $\Psi_{a,b}(t)$ represents the sub-wavelet; *a* is the scale factor; and *b* is the translation factor.

For the sub-wavelet $f(t)\in L^{2}(R)$, the Morlet continuous wavelet analysis is the projection of a function onto the wavelet. The Morlet wavelet is robust to noise and is suitable for dealing with noise in real environmental data, and it could be expressed as follows [33,34,35]:

(12) $$W_{f}(a,b)={\left|a\right|}^{-1/2}\int_{R}f\left(t\right)\Psi^{*}\left(\frac{t-b}{a}\right)dt$$

where $W_{f}(a,b)$ represents the wavelet transform function; $f\left(t\right)$ is a square-integrable function; *a* is the scale factor; *b* is the translation factor; and $\Psi^{*}\left(\frac{t-b}{a}\right)$ is the complex conjugate of the wavelet function $\Psi \left(\frac{t-b}{a}\right)$.

In this study, wavelet analysis was employed to investigate the temporal variation in the impact intensity of each heavy metal source, focusing on the periodicity of these variations. The analysis was performed using the wavelet toolbox in MATLAB 2016. A total of 24 months of continuous data were collected, providing 24 data points for the impact intensity of each source. To eliminate boundary effects, the data were extended by 4 months in both directions, resulting in a dataset of 32 months. The one-dimensional complex continuous wavelet transform was applied to the extended data, and Surfer 14 software was used for data visualization, allowing the identification of temporal patterns in the impact intensity of each source.

## 3. Results and Discussion

### 3.1. The Variations of Particle Size Fraction Load for Heavy Metals

#### 3.1.1. The Characteristics of Particle Size Fraction Load for Different Heavy Metals

**Figure 2:**
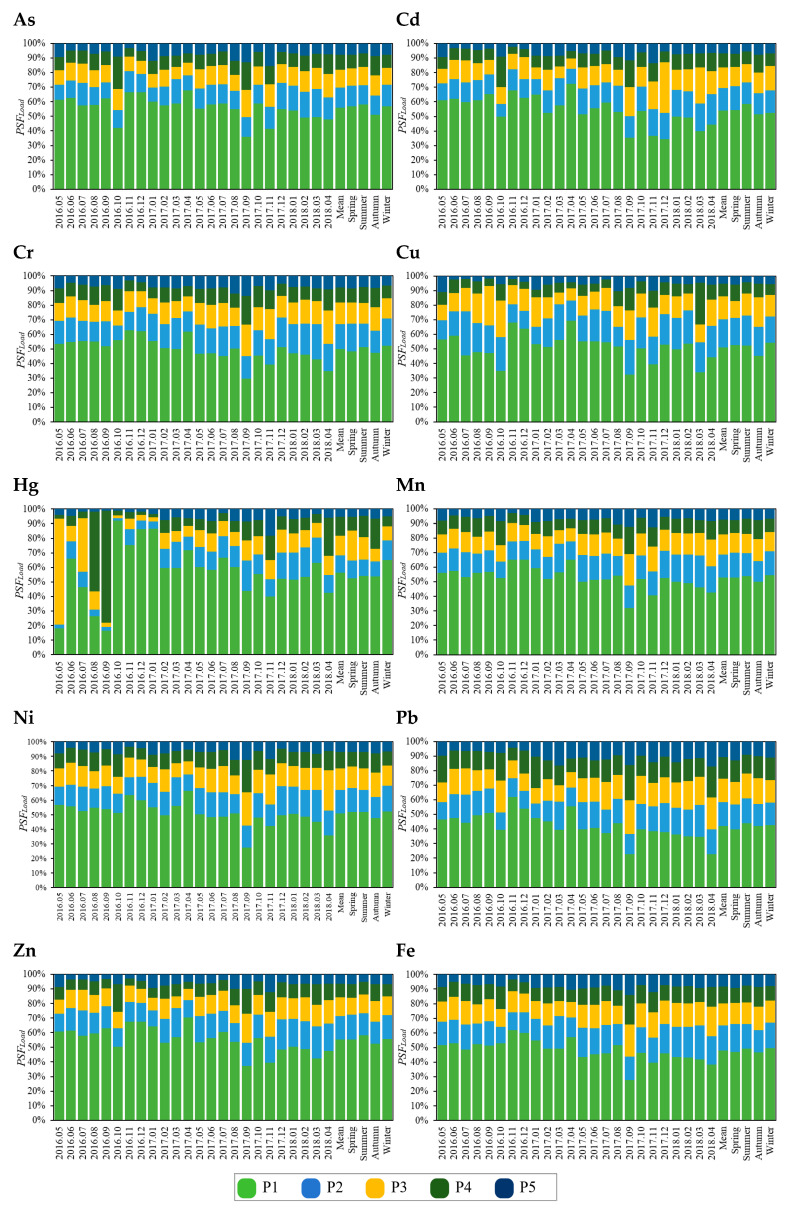
Temporal variation of *PSF_Load_* of different heavy metals in road dust.

For each heavy metal, the load in particle size fraction (0–74 μm, P1) exhibited the highest values across all particle size fractions, reaching as much as 78.38% in some months, whereas particle size fraction (500–1000 μm, P5) consistently recorded the lowest loads, generally below 10% (Figure 2). With the exception of Hg, Ni, and Pb, the load of most heavy metals decreased progressively from P1 to P5. The distribution pattern of heavy metal load is influenced by both the distribution of road dust mass and the heavy metal concentration across particle sizes. Approximately 50% of the total road dust mass was concentrated in P1, whereas P5 accounted for only an average of 8.48% of total mass each month (Figure S1). Excluding Pb, the heavy metal concentration within fractions P1 and P2 was generally higher compared to other particle size fractions. The concentration of heavy metals in P1 was generally higher than that in P5, whereas the mass of road dust in P5 was generally higher than that in P1 (Figures S1 and S2). Therefore, the combination of these two factors might lead to different results for different heavy metals. For Pb, the load pattern was influenced by both a declining mass fraction and an increasing metal concentration as particle size increased, resulting in a slightly higher load in P3 (16.32%) than in P2 (16.14%). Despite this, Pb’s overall load tended to decrease with increasing particle size. It was suggested that the distribution of road dust mass had a more significant effect on heavy metal load than the particle size distribution of heavy metal concentration. The opposite trends in particle size distribution for Pb concentration and road dust mass weakened each other’s effects, causing the smallest difference in Pb load between P1 (42.11%) and P5 (10.74%) among all heavy metals, with only Pb in P5 exceeding 10%. Seasonal variations were also evident in heavy metal loads. For most heavy metals (As, Cd, Cr, Hg, Mn, Ni, Zn, and Fe), the loads in P4 and P5 were higher during autumn than in other seasons. For Cr, Hg, and Ni, the loads in P1 and P2 reached peak values during winter.

#### 3.1.2. The Correlation and Difference of Particle Size Fraction Load Among Fractions

**Figure 3:**
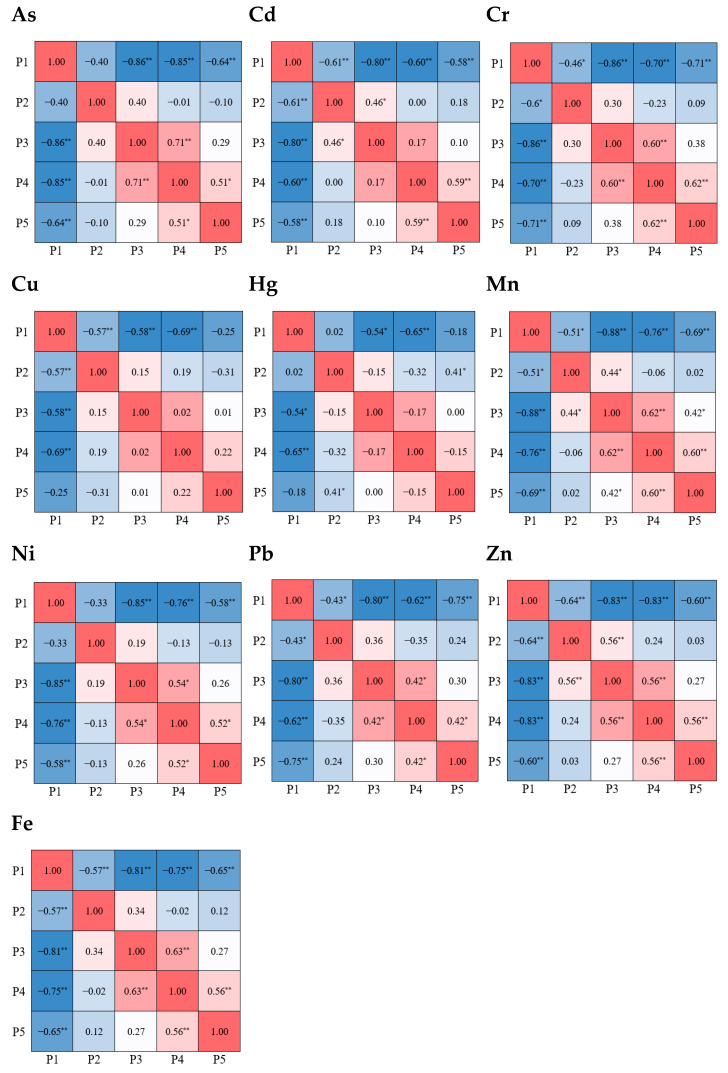
The correlation and difference of *GSF_Load_* between particle size fractions. * indicates *p* < 0.05 and ** indicates *p* < 0.01.

The differences in heavy metal loads among different particle size fractions were compared (Figure 3). For each heavy metal, the loads for different particle size fractions mostly showed significant differences at the 0.01 level (*p* < 0.01). However, differences in heavy metal loads between fractions P2 and P3 were not significant at the 0.01 level, indicating consistency in loads within these fractions. For Pb, differences in loads between fractions P2, P3, and P4 were all non-significant at the 0.05 level, indicating close similarity between these fractions. For Hg, loads in fractions P2, P3, and P4, as well as between P3, P4, and P5, were relatively similar, with significant differences only observed between P1 and other fractions. For all metals, the load in fraction P1 showed significant differences at the 0.01 level compared to other particle size fractions.

**Figure 4:**
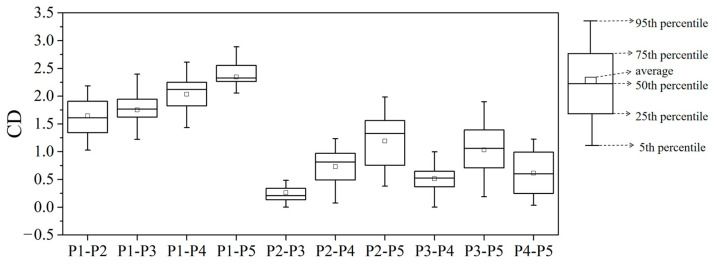
The values of CD among loads of heavy metals in each particle size fraction of road dust. “P2-P3” means a CD value between P2 and P3.

The CD value of heavy metal load between fraction P1 and other particle size fractions was all higher than 0.696 across all months, exceeding the threshold of 0.269, suggesting a low similarity in heavy metal load between P1 and other size fractions (Figure 4). For fractions P2 and P5, the minimum monthly CD value was 0.381, while between P3 and P5, the monthly CD values exceeded 0.269 in most instances. These results indicated that the heavy metal load in fine particle size fractions (P1 to P3) shows limited similarity to that in the larger P5 size fraction. The CD values for heavy metal load between P2 and P3 were the smallest among all size fraction combinations, averaging 0.263 across all months, with a minimum as low as 0.003, highlighting a strong similarity between P2 and P3.

### 3.2. Monthly Variation of Ecological Risk of Heavy Metals Within Particle Size Fractions

#### 3.2.1. Monthly Variation of Ecological Risk of Each Heavy Metal

**Figure 5:**
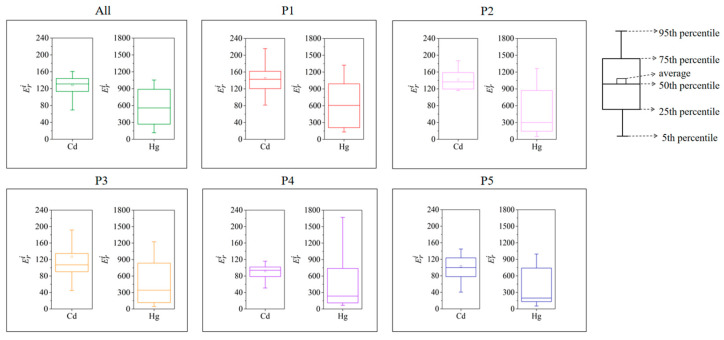
Temporal variations of potential ecological risk associated with Cd and Hg.

For road dust overall, *Er^i^* values for all heavy metals, except for Cd and Hg, remained below 40 throughout the year, suggesting a low ecological risk level for these metals (Figure 5). However, in every month, the lower quartile of *Er^i^* values of Cd exceeded 80, indicating that Cd posed moderate or even higher ecological risk in most months. The lower quartile of *Er^i^* values of Hg was above 160, suggesting that Hg posed a high ecological risk in over 75% of the months. In 70.83% of the months, *Er^i^* values of Hg surpassed 320, reaching extremely high ecological risk levels. These findings indicate that the ecological risks associated with Cd and Hg were significant and warrant prompt intervention measures for effective management.

The ecological risk levels of Cd and Hg showed variations across different particle size fractions. As particle size increased, the proportion of months in which $E_{r}^{i}$ values of Cd and Hg exceeded 80 was decreased. In fraction P1, the $E_{r}^{i}$ values of Cd and Hg exceeded 80 across all months, indicating moderate or higher ecological risk. In contrast, only 75.00% and 87.50% of months in fraction P5 displayed $E_{r}^{i}$ values of Cd and Hg above 80. In fractions P1 to P3, $E_{r}^{i}$ values of Cd exceeded 160 in 16.67% to 29.17% of months, whereas $E_{r}^{i}$ values of Cd in P4 and P5 remained below 160 for all months. For Hg in fraction P1, extreme risk ($E_{r}^{i}$ > 320) was observed in 62.50% of months, whereas this proportion dropped to 33.33% in fraction P5, approximately halving. Overall, the ecological risks associated with Cd and Hg decreased with increasing particle size, highlighting the importance of focusing on finer road dust particles in ecological risk management efforts.

For both the overall road dust and each particle size fraction, the ecological risk of Hg displayed a consistent seasonal trend, with ecological risk peaking in spring and reaching its lowest point in autumn (Table 2). The seasonal variation of *PI_i_* is consistent with the trend of the risk index described above, with the pollution index of Hg peaking in spring and reaching its lowest point in autumn (Table 3). In all fractions, Hg posed extreme risk in spring, while in fraction P3, it only presented moderate risk in autumn, a difference of two risk levels. For all fractions and seasons, severe pollution was posed by Hg. In contrast, the seasonal variation of Cd’s ecological risk differed across particle size fractions. In fraction P1, the ecological risk of Cd reached its maximum in summer (161.99) and its minimum in winter (127.94). In fraction P5, Cd’s ecological risk in summer (91.61) was lower than in winter (112.92). Only in fraction P1 did Cd’s $E_{r}^{i}$ value exceed 160 in summer, indicating high risk, while $E_{r}^{i}$ values remained below 160 for Cd in other fractions and seasons. In fraction P1, *PI_i_* of Cd reached its maximum value (5.40) in summer and its minimum value (4.26) in winter. In fraction P3, *PI_i_* of Cd in summer (4.12) was lower than in winter (5.26). In fraction P4, *PI_i_* of Cd ranged from 2 to 3 during the autumn season, indicating moderate contamination levels. In the other fractions and seasons, the Cd levels were in the severe pollution level. The source apportionment results in Section 3.3.1 of this study provided a plausible explanation for this trend. Traffic emissions were identified as the main source of Cd, with metals from traffic sources showing a tendency to accumulate more in fraction P1, amplifying their impact on Cd levels in this fraction [36]. The influence of traffic emissions was most pronounced in summer, leading to a higher ecological risk for Cd in fraction P1 relative to other seasons. In fraction P5, Cd was less affected by traffic sources, which accounts for the lack of a similar seasonal trend in ecological risk as observed in fraction P1.

#### 3.2.2. *NIRI*-Based Monthly Variation of Integrated Ecological Risk of All Heavy Metals

**Figure 6:**
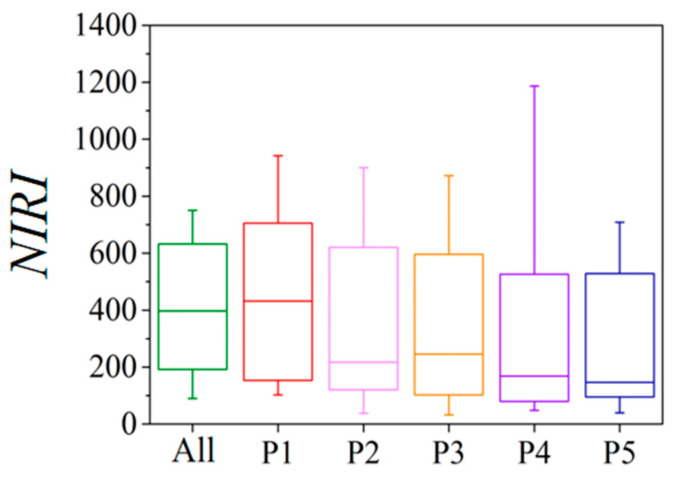
Temporal variations of *NIRI* in each fraction.

For overall road dust, the *NIRI* values were above 80 throughout all months, indicating that the ecological risk level all met or exceeded the moderate-risk threshold (Figure 6). Furthermore, the proportions of months classified as high and extreme risk were 20.84% and 58.33%, respectively. Similar to the ecological risks of each heavy metal, *NIRI* also showed a declining trend with increasing particle size fractions. The monthly *NIRI* values’ quartile distribution showed that fraction P1 exhibited the highest risk, while fractions P4 and P5 showed similar levels and were the lowest among all fractions. Among all particle size fractions, the values of *NIRI* of P1 were above 80 in every month. In contrast, in fractions P4 and P5, months with *NIRI* values above 80 accounted for less than 80% of all months. Notably, 58.33% of months in P1 posed extreme risk, whereas only 29.17% of months reached extreme risk in P5.

Both overall road dust and each particle size fraction showed seasonal variations in integrated ecological risk and integrated pollution index. In spring, the value of *NIRI* was the highest across seasons, with mean *NIRI* values above 320, indicating extreme risk. Conversely, in autumn, the value of *NIRI* was generally lower than in other seasons, with mean *NIRI* values below 320. For fractions P3 and P5, the mean *NIRI* values in autumn were below 160, indicating considerable risk. These findings indicate that ecological risk at a given location may vary by up to two risk levels between different seasons. The value of *NIPI* exceeded three across the different fractions and seasons, indicating a severe level of contamination. In spring, the value of *NIPI* was the highest across seasons, with mean *NIPI* values in the range of 130 to 220.

### 3.3. Identification and Time-Series Analysis of Heavy Metal Sources in Each Fraction

#### 3.3.1. Identification of Heavy Metal Sources in Each Fraction

**Figure 7:**
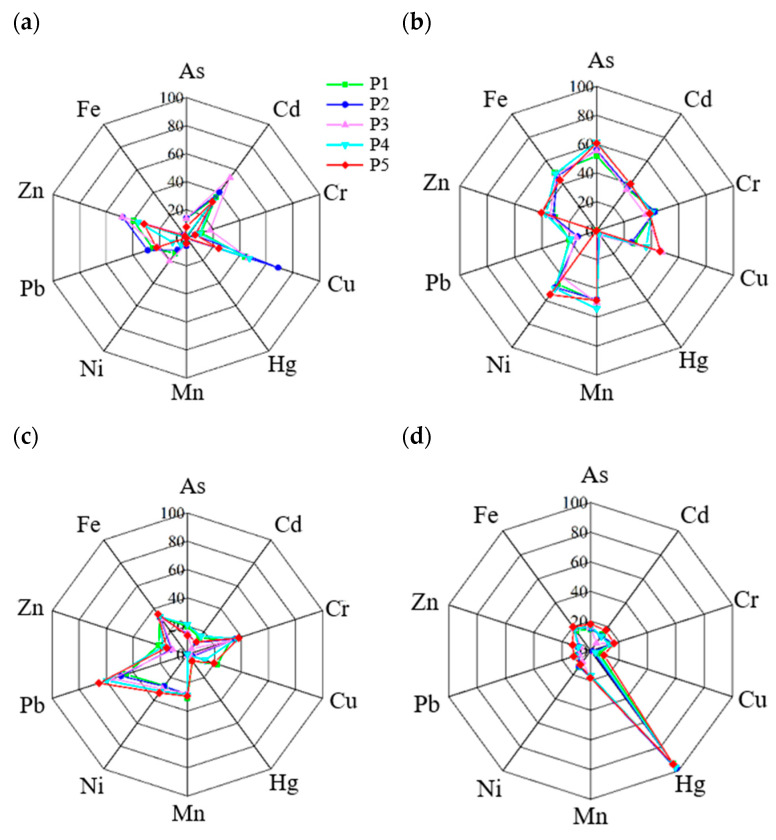
Factor profiles of heavy metals in each fraction based on the ABB three-way model: (**a**) traffic exhaust; (**b**) fuel combustion; (**c**) construction; (**d**) use of pesticides and fertilizers.

Based on the ABB three-way model, heavy metals in each particle size fraction were apportioned into four factors (Figure 7). In the ABB three-way model, Factor 1 was characterized by Cd (31.75–53.11%), Cu (24.41–68.92%), and Zn (31.74–48.09%) for different particle size fractions. Factor 2 was represented by As (51.89–61.43%), Cr (35.67–42.77%), Mn (47.89–54.15%), Ni (40.17–54.83%), and Fe (43.50–49.43%). Factor 3 was primarily associated with Pb, accounting for 45.34–65.35% across particle size fractions, with additional contributions exceeding 30% to Cr in all fractions and approximately 20% to Cu in fractions P2 and P5. Factor 4 was the most dominant source of Hg, with a contribution of 94.22–99.06% for different particle size fractions. The representative heavy metals of each source were similar to those in our previous studies based on the PMF model, and the sources of heavy metals were identified as traffic exhaust, fuel combustion, construction, and use of pesticides and fertilizers [37,38].

The ABB three-way model results indicated that various sources, particularly traffic exhaust and construction, exhibited distinct accumulation patterns among particle size fractions. Contributions from traffic sources to most heavy metals, including Pb, tended to decrease with increasing particle size. The proportion of Cu from traffic exhaust in fraction P5 was only 0.35–0.55 times that in other fractions, suggesting a higher tendency for traffic-related heavy metals to accumulate in finer particles. This pattern aligns with the emission characteristics of motor vehicles, which predominantly emit ultrafine particles [39]. In contrast, contributions from construction, with Pb as a representative element, increased with particle size, rising from 45.34% in P1 to 65.35% in P5. The higher contribution of construction to Pb in P5 than that in P1 might be the main reason for the higher concentration of Pb in P5 than that in P1. The contribution of the use of fertilizer and pesticide to Hg was consistent among particle sizes, with a proportion of higher than 94% in all fractions. Given that fine particles are more prone to resuspension into the atmosphere and to entering water bodies via surface runoff during the rainy season, priority should be given to controlling primary sources of heavy metals in finer road dust fractions [5].

The similarities in the element composition between traffic exhaust and fuel combustion make it challenging for the ABB three-way model to distinguish between the two sources, which is a common issue for related studies. To improve the reliability of source apportionment results, effects might be performed from these points: (1) more detailed information about traffic exhaust and fuel combustion should be collected, which will improve the model’s ability to discriminate these two sources; (2) the ABB three-way model should be applied in conjunction with other source identification techniques (e.g., stable isotope analysis) to improve the accuracy of source differentiation; (3) the algorithms of the ABB three-way model should be optimized with machine learning methods to improve the model’s ability to process complex data.

#### 3.3.2. Time-Series Variation in the Intensity of Heavy Metal Sources in Each Fraction

The impact intensity of each source of heavy metals changed with the seasons (Table 4). Compared to other sources, traffic exhaust exhibited relatively weak seasonal variations, with the highest influence observed in summer (1.18) and the lowest in winter (0.79). This might be attributed to climate factors, such as high temperatures and strong sunlight in summer, as well as fluctuations in tourist numbers [40]. In contrast, fuel combustion sources showed the highest influence in winter, reaching 1.19, which might be due to increased demand for residential heating [41]. The influence of construction peaked in spring and displayed a general increasing trend over the sampling period, possibly due to ongoing construction activities in the surrounding area [42]. Use of pesticides and fertilizers demonstrated the most pronounced seasonal variation, with their influence on heavy metal accumulation peaking at 3.72 in spring, significantly higher than in other seasons. It was suggested that seasonal urban greening and related human activities may substantially affect the contribution of fertilizer and pesticide sources [43].

**Figure 8:**
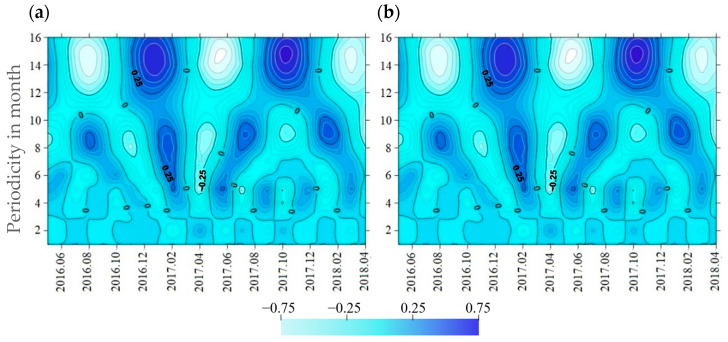
Periodicity of intensities of heavy metal sources: (**a**) traffic exhaust; (**b**) fuel combustion.

Among these four sources, the impact intensity of traffic exhaust and fuel combustion both displayed periodic variations over time (Figure 8). The impact intensity of traffic exhaust showed period cycles of 5–8 months and 10–13 months. The period cycle of 5–8 months exhibited stronger periodicity and produced approximately four oscillations. The impact intensity of traffic exhaust also showed minor fluctuations on a 3-month scale, whereas the fluctuations were negligible compared to the period cycle of 5–8 months. During certain periods, such as between May and October 2016, the cycles of 5–8 months and 10–13 months appeared to blur or even overlap, potentially obscuring the periodic trends in the impact of traffic exhaust. In contrast, the impact of fuel combustion showed stable periodic patterns across three cycles: 3–6 months, 8–10 months, and 12–15 months. The period cycle of 12–15 months was the most pronounced, with approximately two oscillations over the study period. Positive peaks in these oscillations for fuel combustion typically occurred in winter, while negative troughs were observed around summer, reinforcing the impact of urban heating activities in winter. In comparison, the construction and use of pesticides and fertilizers did not exhibit clear periodic trends in their impact intensities over time. This absence of periodicity might suggest that the impact intensities of construction were more closely associated with local construction projects, with limited influence from climatic factors. Limitations in data resolution might also be another reason.

Source apportionment results served as a reference in the control of heavy metal contamination. Reducing heavy metal emissions from fuel combustion sources would be an effective strategy for risk mitigation, especially in winter. Ensuring a clean energy supply and implementing strict pollutant emission controls (such as treatment and disposal facilities) are necessary measures [44,45,46]. For traffic exhaust-related contamination, controlling vehicle numbers and optimizing traffic routes [47], along with promoting technological innovations [48], can help reduce impacts. In addition to managing individual heavy metal sources, it is important to consider particle size and seasonality when adjusting management measures. In spring, it is important to strengthen control over fertilizer and pesticide use, substituting harmful chemicals with less hazardous alternatives to minimize environmental and health impacts [49].

## 4. Conclusions

The highest concentration of heavy metals was observed in the finest particle fraction (P1), whereas the lowest was found in the coarsest class (P5). Except for Hg, Ni, and Pb, the heavy metal concentrations exhibited a clear decreasing trend from P1 to P5. This pattern of heavy metal distribution aligns with the particle size distribution of road dust. However, the influence of Pb content distribution somewhat diminishes the variability in Pb load across different particle classes. Significant differences in heavy metal loads were shown between P1 and all other classes, and only classes P2 and P3 showed some similarities.

Seasonal variations of *NIRI* among different particle size fractions can span up to two risk levels. Fraction P1 posed the highest integrated ecological risk among all fractions, whereas the integrated ecological risk from fractions P4 and P5 was the lowest. The number of months classified as extreme risk for P1 is two times that for P5. The monthly change of integrated ecological risk was similar to that of Hg, suggesting a strong influence of Hg on overall risk.

The main types of heavy metal sources across particle classes are uniform and identified as traffic exhaust, fuel combustion, construction, and use of pesticides and fertilizers. However, the accumulation potential of these sources in particle size fractions varied. Traffic exhaust tended to accumulate in finer particles, with their contribution to Cu in P5 being only 35–55% of that in other fractions. Conversely, construction contributes more to coarser particles, and its contribution to Pb was increased from 45.34% in P1 to 65.35% in P5. For each source, the temporal trend of its impact intensity was consistent among different fractions. The impact intensity of both traffic exhaust and fuel combustion exhibited periodicity throughout the study period. Wavelet analysis indicated that traffic exhaust showed periodicities of 5–8 and 10–13 months. Fuel combustion displayed the strongest periodicity of 12–15 months, peaking in winter. Strategies focused on fuel combustion and traffic exhaust should be adopted, such as ensuring a clean energy supply, implementing strict pollutant emission control, controlling vehicle numbers, and promoting technological innovations. The research framework is referential for studies focused on ecological risk and sources on a city scale, and the results might be influenced by climatic conditions, levels of urbanization, and industrial activities in cities.

## Figures and Tables

**Table 1:** The classifications of *NIRI* and *NIPI*.

Classification	Level
*NIRI* ≤ 40; $E_{r}^{i}$≤ 40	Low risk
40 < *NIRI* ≤ 80; 40 < $E_{r}^{i}$≤ 80	Moderate risk
80 < *NIRI* ≤ 160; 80 < $E_{r}^{i}$≤ 160	Considerable risk
160 < *NIRI* ≤ 320; 160 < $E_{r}^{i}$≤ 320	High risk
*NIRI* > 320; $E_{r}^{i}$> 320	Extreme risk
*NIPI* ≤ 0.7; *PI_i_* ≤ 1	Unpolluted
0.7 < *NIPI* ≤ 1	Warning limit of pollution
1 < *NIPI* ≤ 2; 1 < *PI_i_* ≤ 2	Low polluted
2 < *NIPI* ≤ 3; 2 < *PI_i_* ≤ 3	Moderately polluted
*NIPI* > 3; *PI_i_* > 3	Strongly polluted

**Table 2:** Mean value of ecological risk of heavy metals in different seasons.

Particle Size Fraction	Season	$E_{r}^{i}$										*NIRI*
		As	Cd	Cr	Cu	Hg	Mn	Ni	Pb	Zn	Fe	
P1	Spring	6.03	155.99	3.27	19.37	11,973.66	1.10	6.20	15.37	4.47	1.02	8513.92
	Summer	6.26	161.99	3.14	20.53	550.60	1.00	5.90	12.37	4.93	0.94	393.26
	Autumn	5.84	134.03	3.07	17.13	427.44	0.98	5.69	11.21	4.09	0.96	311.82
	Winter	6.12	127.94	2.95	15.89	805.83	1.04	5.63	11.09	3.71	0.97	574.06
P2	Spring	4.82	149.92	4.15	22.38	10,130.96	1.16	6.50	21.95	4.41	1.32	7211.47
	Summer	5.39	148.79	3.73	31.44	396.43	1.08	6.56	17.76	4.84	1.17	284.71
	Autumn	4.98	130.94	3.28	24.20	233.62	0.95	5.79	13.94	3.98	1.04	184.33
	Winter	5.62	138.28	3.91	19.38	651.95	1.12	6.94	15.14	4.01	1.26	465.01
P3	Spring	4.10	123.50	3.29	14.54	7905.09	0.91	5.80	20.45	3.10	1.04	5622.56
	Summer	4.69	123.48	3.04	20.97	511.46	0.86	5.85	16.48	3.78	1.00	367.30
	Autumn	4.16	103.19	2.83	20.54	151.97	0.80	5.30	13.08	3.05	0.91	123.82
	Winter	4.57	157.93	2.86	15.87	481.09	0.92	5.45	15.26	3.21	1.07	343.82
P4	Spring	3.74	95.02	2.53	18.70	7459.12	0.71	4.62	22.31	2.89	0.87	5302.01
	Summer	3.99	91.55	2.58	11.24	490.32	0.73	5.04	14.73	2.66	0.85	350.58
	Autumn	4.14	87.66	2.54	9.76	945.26	0.72	4.52	11.92	2.45	0.81	674.59
	Winter	4.28	94.61	2.18	9.55	416.63	0.75	4.61	18.30	2.60	0.91	297.31
P5	Spring	4.66	113.45	3.18	11.78	7487.73	0.89	4.61	30.11	2.93	1.10	5324.73
	Summer	4.86	91.61	3.05	9.42	287.19	0.83	5.10	18.61	2.69	0.95	205.40
	Autumn	4.51	91.72	2.49	8.72	175.98	0.70	4.38	13.54	2.41	0.83	128.78
	Winter	5.49	112.92	2.61	10.86	481.31	0.86	25.28	21.40	3.02	1.04	343.65
All particles	Spring	5.15	140.05	3.31	18.35	10,404.70	1.01	5.89	19.61	3.92	1.05	7397.80
	Summer	5.11	125.51	3.07	17.73	483.05	0.91	5.63	14.41	3.81	0.96	344.87
	Autumn	5.06	118.07	2.89	16.58	379.83	0.88	5.31	12.26	3.48	0.92	279.15
	Winter	5.58	129.99	2.95	15.23	661.84	0.98	7.22	13.97	3.52	1.02	471.82

**Table 3:** Mean value of pollution index of heavy metals in different seasons.

Particle Size Fraction	Season	*PI_i_*										*NIPI*
		As	Cd	Cr	Cu	Hg	Mn	Ni	Pb	Zn	Fe	
P1	Spring	0.60	5.20	1.63	3.87	299.34	1.10	1.24	3.07	4.47	1.02	213.25
	Summer	0.63	5.40	1.57	4.11	13.77	1.00	1.18	2.47	4.93	0.94	10.20
	Autumn	0.58	4.47	1.54	3.43	10.69	0.98	1.14	2.24	4.09	0.96	8.25
	Winter	0.61	4.26	1.48	3.18	20.15	1.04	1.13	2.22	3.71	0.97	14.52
P2	Spring	0.48	5.00	2.07	4.48	253.27	1.16	1.30	4.39	4.41	1.32	180.93
	Summer	0.54	4.96	1.87	6.29	9.91	1.08	1.31	3.55	4.84	1.17	8.12
	Autumn	0.50	4.36	1.64	4.84	5.84	0.95	1.16	2.79	3.98	1.04	5.69
	Winter	0.56	4.61	1.95	3.88	16.30	1.12	1.39	3.03	4.01	1.26	11.89
P3	Spring	0.41	4.12	1.64	2.91	197.63	0.91	1.16	4.09	3.10	1.04	141.26
	Summer	0.47	4.12	1.52	4.19	12.79	0.86	1.17	3.30	3.78	1.00	9.64
	Autumn	0.42	3.44	1.41	4.11	3.80	0.80	1.06	2.62	3.05	0.91	4.21
	Winter	0.46	5.26	1.43	3.17	12.03	0.92	1.09	3.05	3.21	1.07	9.17
P4	Spring	0.37	3.17	1.26	3.74	186.48	0.71	0.92	4.46	2.89	0.87	133.77
	Summer	0.40	3.05	1.29	2.25	12.26	0.73	1.01	2.95	2.66	0.85	9.14
	Autumn	0.41	2.92	1.27	1.95	23.63	0.72	0.90	2.38	2.45	0.81	17.29
	Winter	0.43	3.15	1.09	1.91	10.42	0.75	0.92	3.66	2.60	0.91	7.76
P5	Spring	0.47	3.78	1.59	2.36	187.19	0.89	0.92	6.02	2.93	1.10	134.49
	Summer	0.49	3.05	1.52	1.88	7.18	0.83	1.02	3.72	2.69	0.95	5.62
	Autumn	0.45	3.06	1.25	1.74	4.40	0.70	0.88	2.71	2.41	0.83	3.69
	Winter	0.55	3.76	1.31	2.17	12.03	0.86	5.06	4.28	3.02	1.04	9.55
All particles	Spring	0.60	5.20	1.63	3.87	299.34	1.10	1.24	3.07	4.47	1.02	213.25
	Summer	0.63	5.40	1.57	4.11	13.77	1.00	1.18	2.47	4.93	0.94	10.20
	Autumn	0.58	4.47	1.54	3.43	10.69	0.98	1.14	2.24	4.09	0.96	8.25
	Winter	0.61	4.26	1.48	3.18	20.15	1.04	1.13	2.22	3.71	0.97	14.52

**Table 4:** Impact intensity of heavy metal sources in different seasons.

Seasons	Traffic Exhaust	Fuel Combustion	Construction	Use of Pesticides and Fertilizers
Spring	1.15	0.52	1.41	3.72
Summer	1.18	1.12	0.91	0.10
Autumn	0.88	1.16	0.68	0.05
Winter	0.79	1.19	1.00	0.13

## Data Availability

Data available on request due to restrictions.

## Supplementary Materials

The following supporting information can be downloaded at: https://www.mdpi.com/article/10.3390/toxics13010040/s1, Figure S1: Temporal variation of concentrations of heavy metals in different particle size fractions; Figure S2: Distribution of mass of road dust in different particle size fractions.
