# Supplementary material for: Particle Size-Dependent Monthly Variation of Pollution Load, Ecological Risk, and Sources of Heavy Metals in Road Dust in Beijing, China
Source: Toxics. 2025 Jan 7;13(1):40. doi: 10.3390/toxics13010040 (PMC11769404; doi:10.3390/toxics13010040)
Supplement: Supplementary file 1 [file toxics-13-00040-s001.zip › toxics-3380443-supplementary.pdf]

Particle Size-Dependent Monthly Variation of Pollution Load, Ecological Risk, and Sources of Heavy Metals in Road Dust in Beijing, China

Supporting information

List

Figure S1 Temporal variation of concentrations of heavy metals in different particle size fractions

Figure S2 Distribution of mass of road dust in different particle size fractions

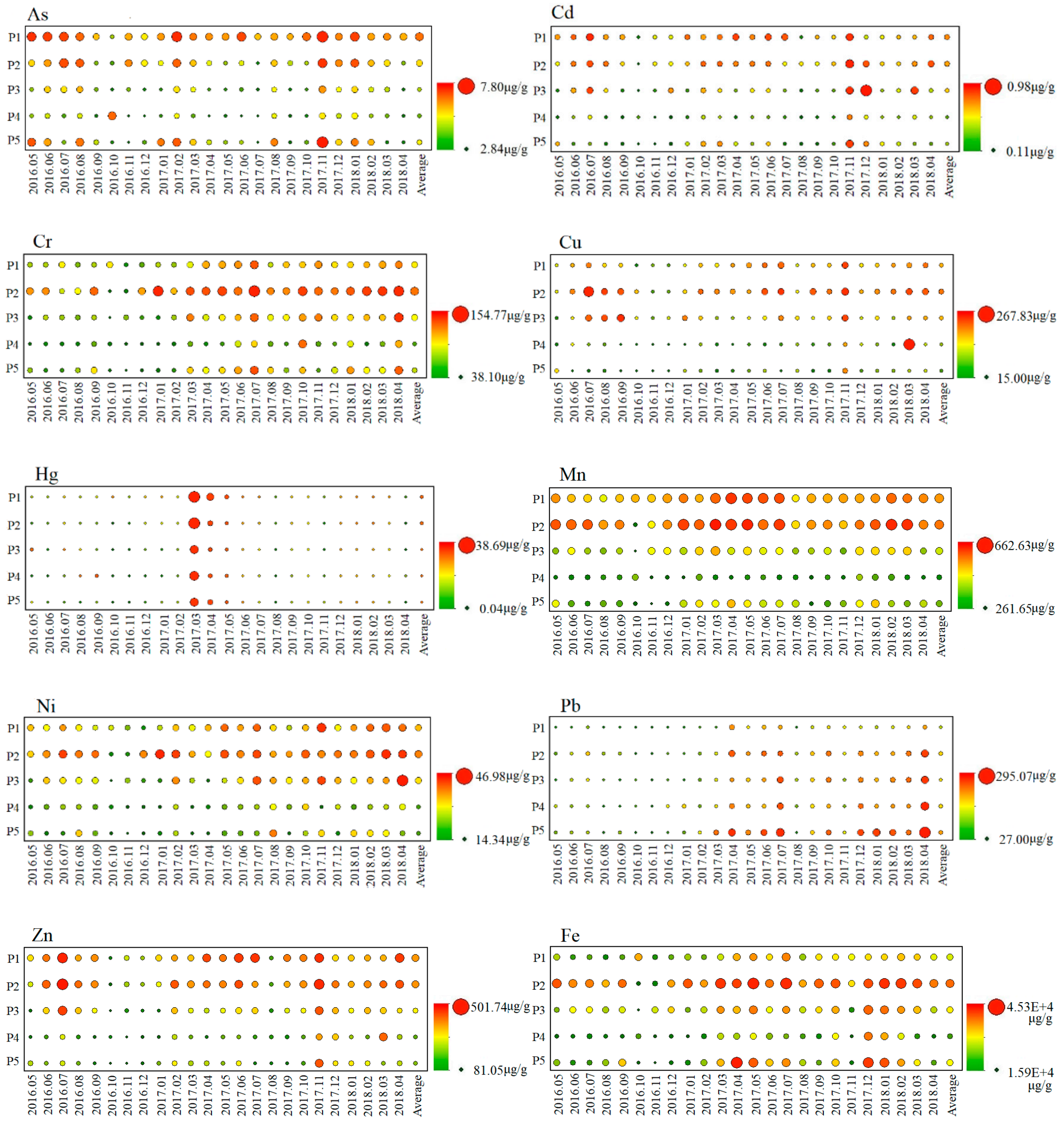

Figure S1 Temporal variation of concentrations of heavy metals in different particle size fractions

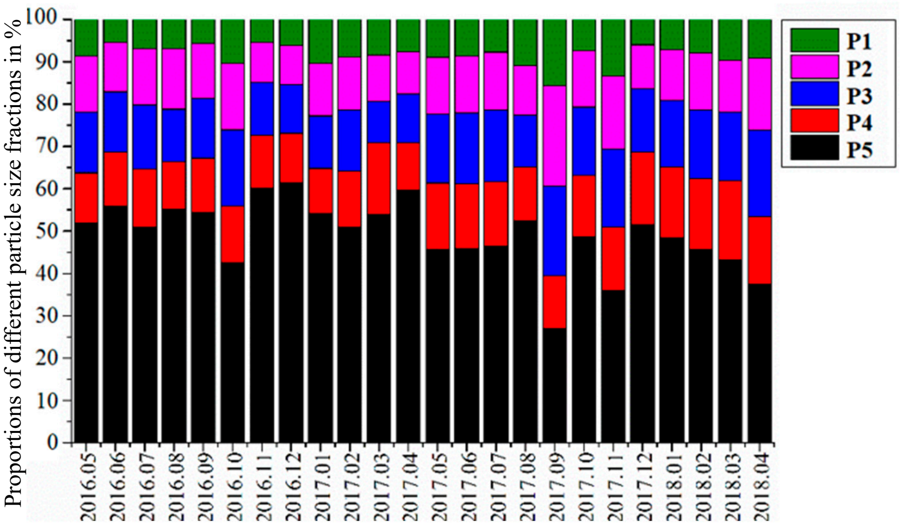

Figure S2 Distribution of mass of road dust in different particle size fractions
